# Glymphatic System Efficiency, Choroid Plexus Volume, and Lateral Ventricle Volume Variations in Normal Individuals and Throughout the Progression of Alzheimer's Disease

**DOI:** 10.1002/alz.083874

**Published:** 2025-01-09

**Authors:** Moto Nakaya, Koji Kamagata, Kaito Takabayashi, Wataru Uchida, Akifumi Hagiwara, Andica Christina, Akihiko Wada, Toshiaki Taoka, Shinji Naganawa, Osamu Abe, Shigeki Aoki

**Affiliations:** ^1^ The University of Tokyo, Tokyo Japan; ^2^ Juntendo University, Tokyo Japan; ^3^ Nagoya University, Nagoya Japan

## Abstract

**Background:**

Alzheimer’s disease (AD) progression is often characterized by the accumulation of amyloid and tau proteins, which can be linked to impaired brain clearance mechanisms, including the glymphatic system. Our research evaluates noninvasive MRI‐based indicators of brain clearance functionality, such as choroid plexus volume (CPV), lateral ventricular volume (LVV), and the perivascular space diffusivity index (ALPS index), throughout various stages of AD.

**Method:**

We analyzed MRI data measuring CPV, LVV, and ALPS index from participants categorized as amyloid‐beta (Aβ)‐ negative healthy controls (HC), Aβ‐positive HC, Aβ‐negative subjective cognitive decline (SCD), Aβ‐positive SCD, mild cognitive impairment (MCI), and AD, using the Alzheimer's Disease Neuroimaging Initiative database. A General Linear Model was used for cross‐group comparisons of MRI metrics. Additionally, partial Spearman's Rank Correlation tests were conducted to investigate associations between MRI measures, neuropsychological assessments, and CSF biomarker levels within each group.

**Result:**

The participant pool comprised 21 Aβ‐negative cognitively healthy individuals, 10 Aβ‐positive HCs, 21 patients with Aβ‐negative SCD, 9 with Aβ‐positive SCD, 19 MCI patients, and 26 AD patients. We observed increased CPV and LVV and decreased ALPS index in advanced stages of the disease. Notably, the ALPS index was significantly reduced in Aβ‐positive HCs compared to Aβ‐negative ones. In SCD subjects and across the AD spectrum, correlations were found between these MRI indicators of brain clearance and both tau protein levels and cognitive performance metrics.

**Conclusion:**

Our findings suggest that MRI‐detected alterations in brain clearance metrics are associated with Aβ deposition and can be identified early in SCD that is linked to AD pathology.

